# Supplementary material for: A potent antimalarial benzoxaborole targets a Plasmodium falciparum cleavage and polyadenylation specificity factor homologue
Source: Nat Commun. 2017 Mar 6;8:14574. doi: 10.1038/ncomms14574 (PMC5343452; doi:10.1038/ncomms14574)
Supplement: Supplementary Information — Supplementary Figures and Supplementary Tables [file ncomms14574-s1.pdf]

Supplementary Tables

Supplementary Table 1. Susceptibility to AN3661 of field isolates, laboratory strains, and parasites selected or modified *in vitro*.

Parasite	IC_{50} values (nM)	Number of assays	IC_{50} fold change	PfCPSF3	# copies of *pfmdr1*
Field isolate 1	61 (36-104)	1	N/A	ND	ND
Field isolate 2	152 (110-211)	1	N/A	ND	ND
Field isolate 3	25 (18-35)	1	N/A	ND	ND
Field isolate 4	13 (9-19)	1	N/A	ND	ND
Field isolate 5	73 (55-96)	1	N/A	ND	ND
Field isolate 6	45 (33-61)	1	N/A	ND	ND
Field isolate 7	53 (36-78)	1	N/A	ND	ND
Field isolate 8	13 (10-17)	1	N/A	ND	ND
Field isolate 9	14 (9-20)	1	N/A	ND	ND
Field isolate 10	29 (27-31)	1	N/A	ND	ND
Field isolate 11	162 (97-271)	1	N/A	ND	ND
Field isolate 12	145 (121-173)	1	N/A	ND	ND
3D7 [a]	34.3 ± 2.9	3	1	ND	1
W2 [a]	31.2 ± 3.0	13	1	WT	1
W2-R1 [a]	101 ± 20.0	6	3	WT	3
W2-R2 [a]	414 ± 31.9	3	13	WT	4
W2-R3 [a]	722 ± 85.0	6	23	D470N	4
W2-R3^{rev} [a]	23.4 ± 3.2	3	1	WT	N.D.
W2-R4 [a]	6240 ± 870	6	200	H36/D470N	4
W2-R4^{rev} [a]	2970 ± 133	3	95	H36/D470N	N.D.
W2-R5 [a]	15300 ± 2940	2	491	ND	N.D.
W2-R5^{rev} [a]	2270 ± 284	3	73	H36/D470N	3
Dd2 [a, b]	22.2 ± 3.0	27	1	WT	3
Dd2-R1 [a]	395 ± 48.8	3	18	D470N	3
Dd2-R2 [a]	921 ± 100	3	41	D470N	3

Dd2-Ra [b]	338 ± 38.4	9	16	T406I	3
Dd2-Rb [b]	623 ± 83.4	11	29	Y408S	3
Dd2-Rc [b]	225 ± 32.8	3	11	T409A	3
Dd2 transf. C4 [Φ b]	563 ± 123	5	25	Y408S	3
Dd2 transf. C4 cl.4 [Φ b]	1550 ± 1070	2	70	Y408S	3
Dd2 transf. C4 cl.7 [Φ b]	523 ± 179	2	24	Y408S	3
Dd2 transf. D3 [Φ b]	1110 ± 229	9	50	Y408S	3
Dd2 transf. E1 [Φ b]	272 ± 94.9	4	12	T406I	3
Dd2 transf. F1 [Φ b]	312 ± 96.2	4	14	T406I	3
Dd2 transf. F3 [Φ b]	300 ± 126	4	14	T406I	3

For field isolates, *ex vivo* IC_{50}s were determined from single assays using a histidine-rich protein-2 ELISA. 95% confidence intervals (in parentheses) describe precision of IC_{50} determined from the curve fit. For laboratory strains, each assay was done in duplicate, and values are shown as mean IC_{50} ± S.E.M. [a]IC_{50}s were calculated from 48 h dose-response data measured by flow cytometric analysis of parasites stained with YOYO-1 dye. [b]IC_{50}s were calculated from 72 h dose-response data measured by flow cytometric analysis of parasites stained with SYBR Green I and Mitotracker Deep Red. [Φ]Dd2 parasites transfected with PFCPSF3 Y408S or T406I, as described in Fig. 5. WT: wild-type. ND: not determined. N/A: not applicable.

Supplementary Table 2. Sensitivity of laboratory strains to AN3661 and standard antimalarials.

	AN3661	Chloroquine	Atovaquone	Artemisinin
Parasite	IC_{50} (nM)	IC_{50} (nM)	IC_{50} (nM)	IC_{50} (nM)
3D7	52 ± 23	<10 ± 1	2 ± 1	29 ± 13
K1	20 ± 3	411 ± 131	2 ± 0.5	19 ± 10
Dd2	39 ± 21	425 ± 163	2 ± 1	31 ± 11
HB3	56 ± 4	14 ± 1	1 ± 0.5	28 ± 6
FCR3	30 ± 3	176 ± 29	1370 ± 392	14 ± 1
TM90C2B	28 ± 1	291 ± 39	>5000	30 ± 7

IC_{50}s were calculated from 48 h dose-response data measured by [^{3}H]-hypoxanthine incorporation, with 2-3 replicates for each concentration with each strain. Values indicate mean ± S.D., shown in nM. Fold change compared to 3D7 parasites, which are multi-drug sensitive.

Supplementary Table 3. Activity of AN3661 against mammalian cell lines.

Cell line	Species	Cell lineage	CC_{50} values (μM)
Jurkat	*Homo sapiens*	T lymphocyte	60.5
HeLa	*Homo sapiens*	Cervical endothelial cells	>100
HepG2	*Homo sapiens*	Hepatocyte	>30
L929	*Mus musculus*	Fibroblast	>48.5
Vero	*Cercopithecus aethiops*	Kidney epithelial cells	>20

Cytotoxic activity of AN3661 against human, murine, or simian cell lines was measured in 3-day colorimetric assays using the tetrazolium dye MTT 3-(4,5-dimethylthiazol-2-yl)-2,5-diphenyltetrazolium bromide. CC_{50} values are concentrations at which 50% cytotoxicity was observed. For each cell line assays included 2-3 replicates per concentration. All cells were from ATCC (catalogue numbers: Jurkat, TIB-152; HeLa, CCL-2; HepG2, HB-8065; L929, CCL-1; Vero, CCL-81).

Supplementary Table 4. Time to recrudescence in AN3661- and atovaquone-pressured *Plasmodium falciparum* Dd2.

Compound	Selection pressure (nM)	Initial inoculum	Outcome	
AN3661	60	1×10^8	3 of 3	(19, 19, 19)
		1×10^7	1 of 3	(23)
		1×10^6	2 of 3	(45, 56)
Atovaquone	5	1×10^8	6 of 6	(24, 29, 36, 45, 45, 50)
		1×10^7	2 of 6	(45, 45)
		1×10^6	1 of 6	(45)

Outcome shown as number of flasks in which parasites recrudesce out of total numbers of flasks subjected to drug pressure. In brackets, day on which parasites could be microscopically identified.

Supplementary Table 5. Susceptibility to chloroquine in parasites selected for resistance to AN3661.

Parasite	PfCPSF3	*pfmdr1* copy #	IC_{50} (nM)	IC_{50} fold change	Number of assays
W2	WT	1	45.8 ± 2.5	1.0	3
W2-R1	WT	3	34.4 ± 2.0	0.8	3
W2-R2	WT	4	19.8 ± 3.0	0.4	6
W2-R3	D470N	4	11.9 ± 1.8	0.3	6
W2-R3^{rev}	WT	ND	31.6 ± 4.5	0.7	3
W2-R4	H36Y/D470N	4	15.6 ± 2.2	0.3	6
W2-R5	H36Y/D470N	3	15.8 ± 4.0	0.3	3
Dd2	WT	3	53.6 ± 4.4	1.0	3
Dd2-R1	D470N	3	21.5 ± 1.4	0.4	3
Dd2-R2	D470N	3	16.1 ± 2.2	0.3	3

IC_{50} values were calculated from 48 h dose-response data measured by flow cytometry of parasites stained with YOYO-1. Values indicate mean ± S.E.M., shown in nM. Fold change compared to parental lines. ND: not determined.

Supplementary Table 6.

Table S6. Whole-genome sequencing.

Parasite line	W2 (WT)	W2-R1	W2-R2	W2-R3	W2-R4	W2-R5	Dd2 (WT)	Dd2-R1	Dd2-R2	Dd2-Ra cl.1	Dd2-Ra cl.2	Dd2-Rb cl.1	Dd2-Rb cl.2
Genome coverage (x)	19	152	25	24	161	*106*	106	174	62	110	120	124	105
% covered by 5 or more reads	82	84	65	68	78	*81*	75	77	78	79	76	77	75
SNPs identified													
Top SNPs (200 per chromosome)		2834	2800	2802	2836	2805		2839	2806	2804	2804	2803	2802
real SNPs*		1506	819	1450	1544	1445		1400	1388	1035	1003	1055	1070
SNPs covered by 5 or more reads		1506	819	1178	1544	1443		1400	1386	1035	1003	1055	1070
intergenic		999	463	614	971	906		946	889	585	590	594	617
Intronic		150	71	71	151	135		114	102	120	100	121	127
< 80% coverage has SNP		351	272	463	411	398		329	372	313	298	319	309
Hypervariable genes		1	4	18	7	1		8	17	4	6	8	7
synonymous		0	2	9	0	0		0	3	5	4	4	3
nonsynonymous		5	7	3	4	3		3	3	8	5	9	7

*compared to 3D7 ref genome and parental genome (W2 or Dd2)

Supplementary Table 7. Parasites transfected with plasmids expressing *pfcpsf3* SNPs.

Name	PfCPSF3 mutation	Selection strategy	Day post-transfection parasites are observed	Number of binding site mutations	Sequence (mutations in lower case)
Dd2 transf. F1	T406I	AN3661	16	3	tCCgTGcGTTATTATGGCTTCCC
Dd2 transf. F3	T406I	AN3661	21	3	tCCgTGcGTTATTATGGCTTCCC
Dd2 transf. E1	T406I	AN3661	17	9	tCCgTGcGTgATcATGGCcagtC
Dd2 transf. C4	Y408S	WR+BSD	34	3	tCCgTGcGTTATTATGGCTTCCC
Dd2 transf. D3	Y408S	AN3661	19	9	tCCgTGcGTgATcATGGCcagtC

Dd2 was co-transfected with a plasmid expressing Cas9 and h*dhfr*, and a plasmid expressing PfCPSF3 T406I or Y408S and blasticidin *S*-deaminase. Transfected parasites were maintained in media containing 170 nM AN3661 or media containing 2.5 nM WR99210 and 2µg/ml blasticidin. Parasites that incorporated the PfCPSF3 mutations also incorporated either three or nine silent binding site mutations. The day at which parasites were first microscopically observed is noted. WR: WR99210. BSD: blasticidin.

Supplementary Table 8. Primers used in this study.

Primer	Sequence	Lab identification	Assay
1	AATGATACGGCGACCACCGA	5Sol1_20	PCR amplification for WGS
2	CAAGCAGAAGACGGCATACG	5Sol2_21	PCR amplification for WGS
3	TAAATAGTTTCCTTTGACAAATATTAA	CPSF_1F	PCR amplifcation for PF3D7_1438500
4	TTAATAGAAGAAAAACAATATTTATCT	CPSF_1R	PCR amplifcation for PF3D7_1438500
5	TATAATTCCTTCAATTTGATTATTTAC	CPSF_2F	PCR amplifcation for PF3D7_1438500
6	ATATATGAACGTTGTTAATAAGAATAA	CPSF_2R	PCR amplifcation for PF3D7_1438500
7	TAAATGTTTCATAAATACATAATGATT	CPSF_3F	PCR amplifcation for PF3D7_1438500
8	AAAAAGAAATAGGAAAAAGGATTTA	CPSF_3R	PCR amplifcation for PF3D7_1438500
9	TCCATGTCAACAAAAACTTGTACA	CPSF98_F	Dideoxy sequencing for PF3D7_1438500
10	TGCTGATGTGCTTACTGATCA	CPSF499_R	Dideoxy sequencing for PF3D7_1438501
11	ACGGACTTGATAACCAATTAAT	CPSF585_F	Dideoxy sequencing for PF3D7_1438502
12	TCTGTAAAGAATGAAATGGGTGA	CPSF996_R	Dideoxy sequencing for PF3D7_1438503
13	ACCTTCTTTACAAATTCACCACA	CPSF1610_F	Dideoxy sequencing for PF3D7_1438504
14	GGCTTCCCCTGGTATGCTAC	CPSF1501_R	Dideoxy sequencing for PF3D7_1438505
15	TGTTTCTATTAAATCCATAGTTT	CPSF2086_F	Dideoxy sequencing for PF3D7_1438506
16	TCCCAACGAAATAAGAGAATCA	CPSF1093_F	Dideoxy sequencing for PF3D7_1438507
17	TGTTTCTATTAAATCCATAGTTT	CPSF2086_F	Dideoxy sequencing for PF3D7_1438508
18	TCACTAGCACCCCCTAGACA	CPSF2591_F	Dideoxy sequencing for PF3D7_1438509
19	AGGTGCCTGTATGTTTTTAGTAGA	CPSF2017_R	Dideoxy sequencing for PF3D7_1438510
20	TGCATCTATAAAACGATCAGACAAA	*pfmdr1*-Forward	*pfmdr1* qPCR
21	TCGTGTGTTCCATGTGACTGT	*pfmdr1*-Reverse	*pfmdr1* qPCR
22	6FAM-TTTAATAACCCTGATCGAAATGGAACCTTTG-TAMRA	*pfmdr1*-probe	*pfmdr1* qPCR
23	TGATGTGCGCAAGTGATCC	*β-tubulin*-Forward	*b-tubulin* qPCR
24	TCCTTTGTGGACATTCTTCCTC	*β-tubulin*-Reverse	*b-tubulin* qPCR
25	VIC-TAGCACATGCCGTTAAATATCTTCCATGTCT-TAMRA	*β-tubulin*-probe	*b-tubulin* qPCR
26	ACCCATGCTGATTAGACAATT	28SrRNA_3694F	Northern blots
27	TCGTCTACGATTTGGGCT	28SrRNA_4016R	Northern blots
28	TGAACTTGCTGAATTTGGTA	1cyspxn_147F	Northern blots
29	ATACAGCATTTGTCTCCTTC	1cyspxn_557R	Northern blots
30	GAGATCCAGGAAGAGTCGAC	PNP_F	Northern blots
31	AAATCCCCTTCGTCCCATTT	PNP_R	Northern blots
32	TCTCAAAAATTGATGAAGCC	FP2A_278F	Northern blots
33	CACCACTATGTAATCTCCAA	FP2A_817R	Northern blots
34	ATTGGGAAGCCATAATAACACAT	p5146	*pfcpsf3* gRNA
35	AAACATGTGTTATTATGGCTTCC	p5147	*pfcpsf3* gRNA
36	TCCGTGCGTGATCATGGCCAGTCCTGGTATGCTACAAAATGGAATAT	p5152	*pfcpsf3* gRNA binding site protection
37	TTATTGTCTTGATATAAATAACTAGATATTGATTCAAG	p5153	*pfcpsf3* gRNA binding site protection
38	TCCGTGCGTTATTATGGCTTCCCCTGGTATGCTACAAAATGGAATAT	p5379	*pfcpsf3* gRNA binding site protection
39	GGGCCCGAAAATAATGATAATGGTGATGG	p5150	*pfcpsf3* donor
40	GGATCCTTAATAGTTTGATCAATCATGGC	p5151	*pfcpsf3* donor
41	TAACTTTCAATTGTAAAAGAGACC	p5573	PCR *pfcpsf3* (5' UTR)
42	TGTTTTAATAGATAGGTATAACCC	p5574	PCR *pfcpsf3* (3' UTR)
43	TCAGCTTATTCTTATTAACAACG	p4808	*pfcpsf3* sequencing
44	AATATAGTATGTCTAGGGGGTGC	p4364	*pfcpsf3* sequencing
45	AATTACAGAACCACAAAATGTACC	p5563	*pfcpsf3* sequencing
46	TTTTACTAATGTCGAGAGAATCTC	p5564	*pfcpsf3* sequencing
47	CACATGGGTAGTAGGGACTCC	p5566	*pfcpsf3* sequencing

Supplementary Figures

Supplementary Figure 1.

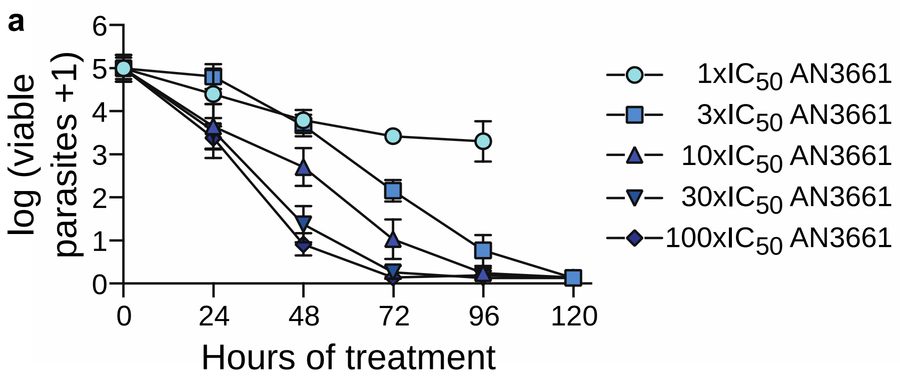

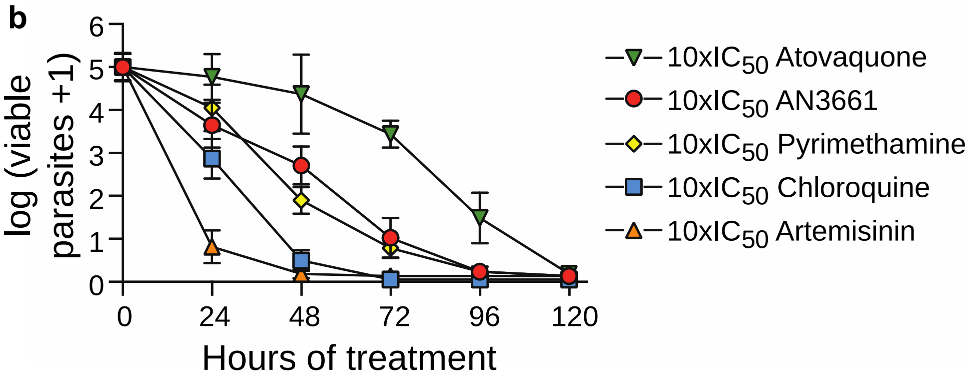

Supplementary Fig. 1. Reduction of *P. falciparum in vitro*. Parasites treated with (**a**) 1×, 3×, 10×, 30×, or 100× IC_{50} AN3661 for 120 hours, or with (**b**) 10× IC_{50} atovaquone, AN3661, pyrimethamine, chloroquine, or artemisinin for 120 hours. Every 24 hours, samples were collected, the drug was washed out, fresh erythrocytes were added, and culture was continued. Parasitemias were determined at the indicated time points by [^{3}H]-hypoxanthine incorporation. Each curve represents the result of 4 independent serial dilutions. Error bars show SD.

Supplementary Figure 2.

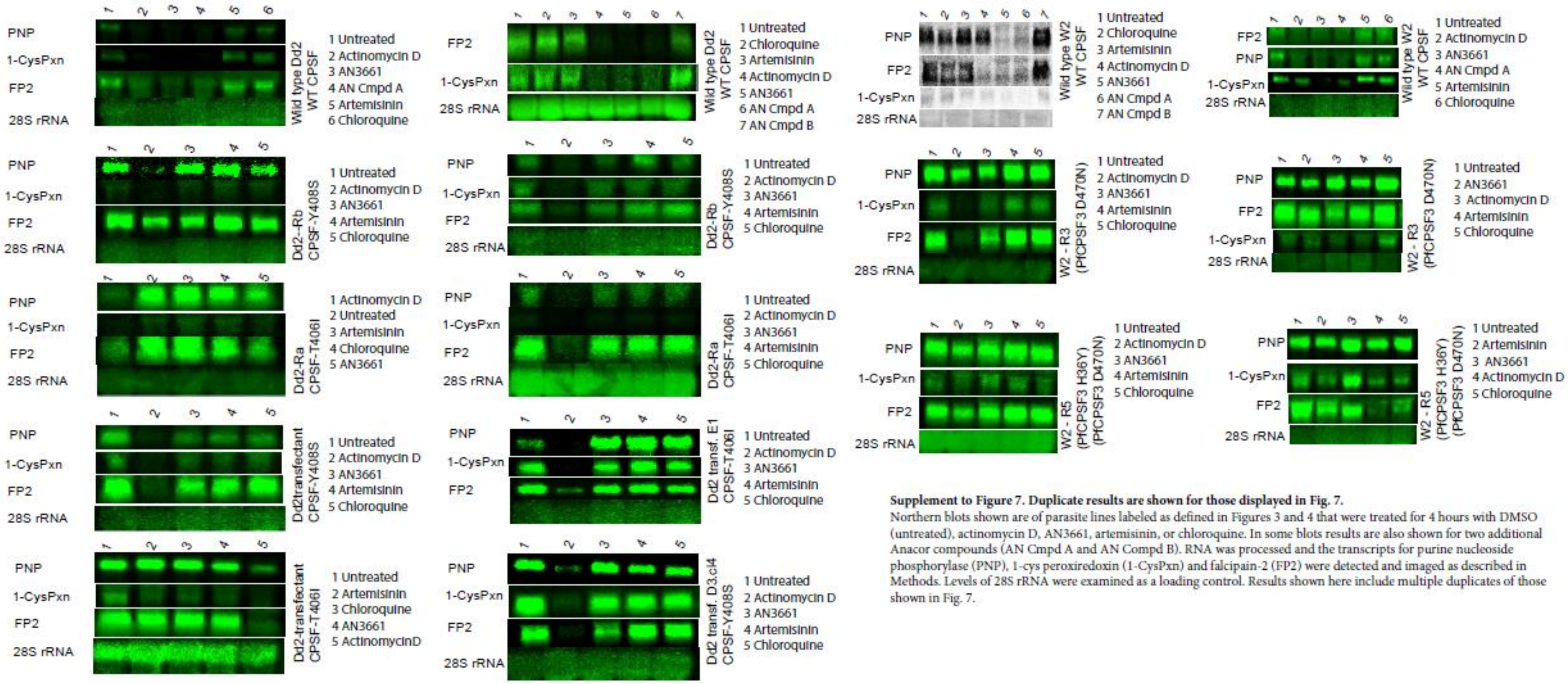

Supplement to Figure 7. Duplicate results are shown for those displayed in Fig. 7.
Northern blots shown are of parasite lines labeled as defined in Figures 3 and 4 that were treated for 4 hours with DMSO (untreated), actinomycin D, AN3661, artemisinin, or chloroquine. In some blots results are also shown for two additional Anacor compounds (AN Cmpd A and AN Compd B). RNA was processed and the transcripts for purine nucleoside phosphorylase (PNP), 1-cys peroxiredoxin (1-CysPxn) and falcipain-2 (FP2) were detected and imaged as described in Methods. Levels of 28S rRNA were examined as a loading control. Results shown here include multiple duplicates of those shown in Fig. 7.

Supplementary Figure 3. Uncropped images used to create Figure 7

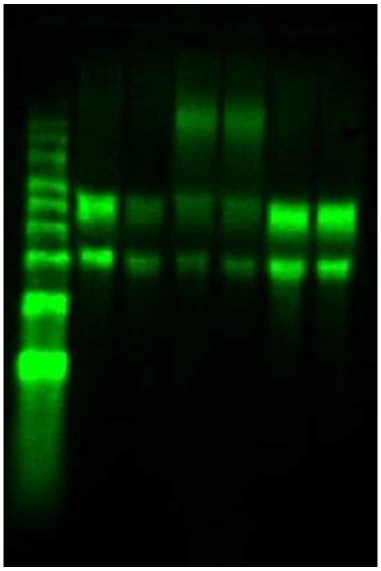

W2 WT-FP2-CysPxn

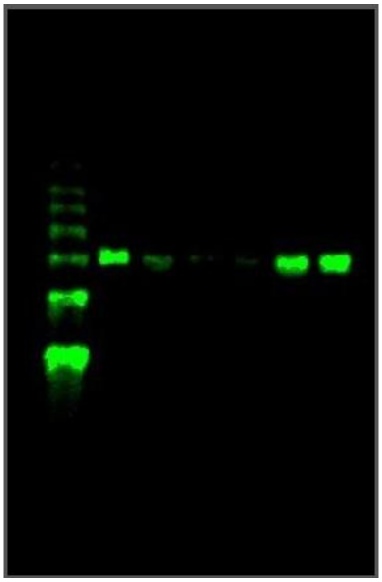

W2 WT-FP2-PNP

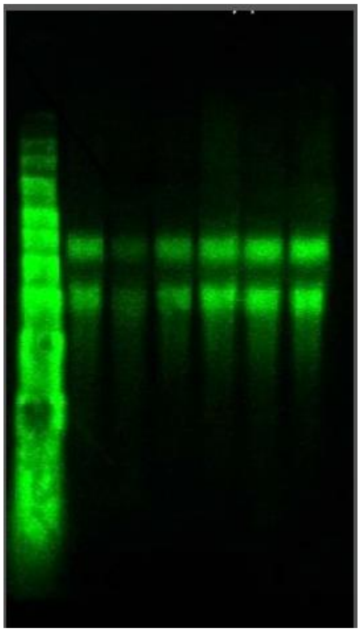

W2 R2-FP2-CysPxn

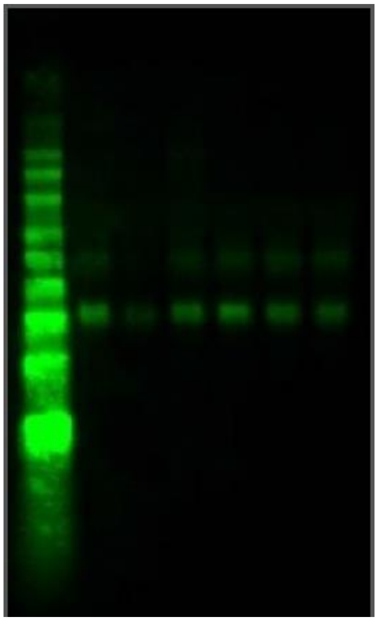

W2 R2-PNP

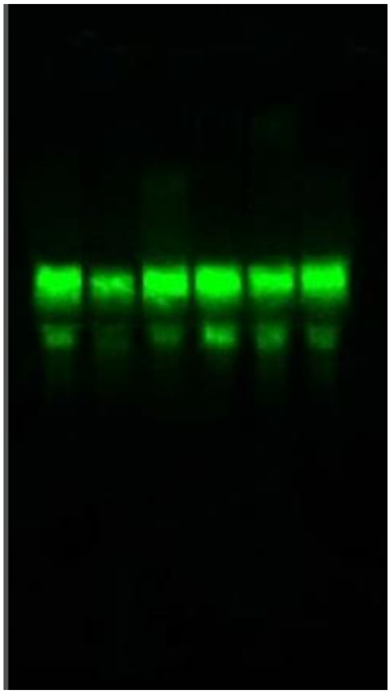

W2 R3-FP2-CysPxn

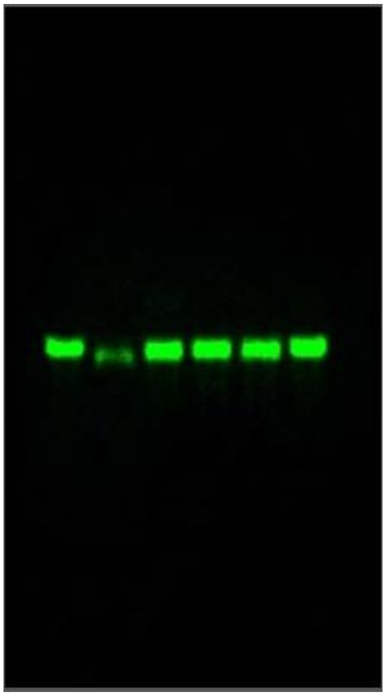

W2 R3-PNP

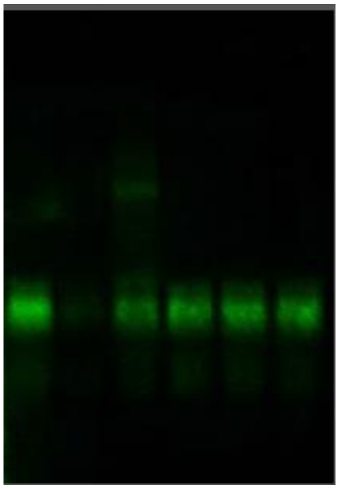

W2 R5-CysPxn

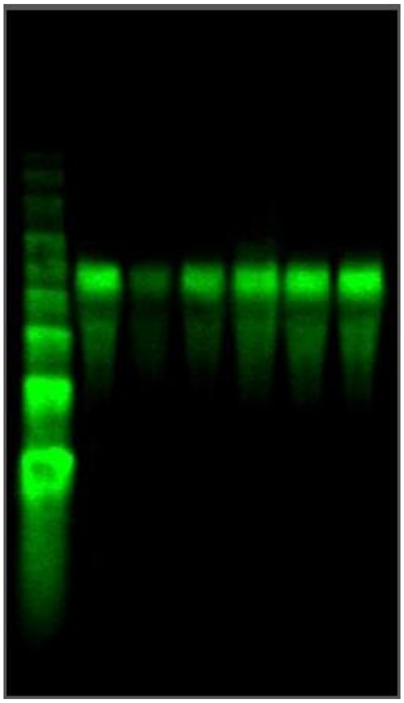

W2 R5-FP2

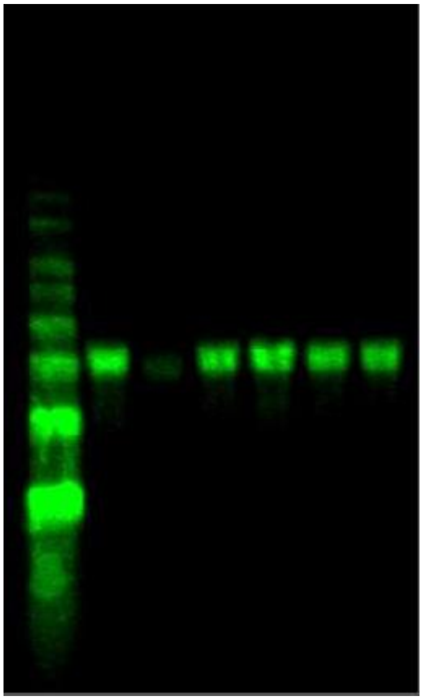

W2 R5-PNP

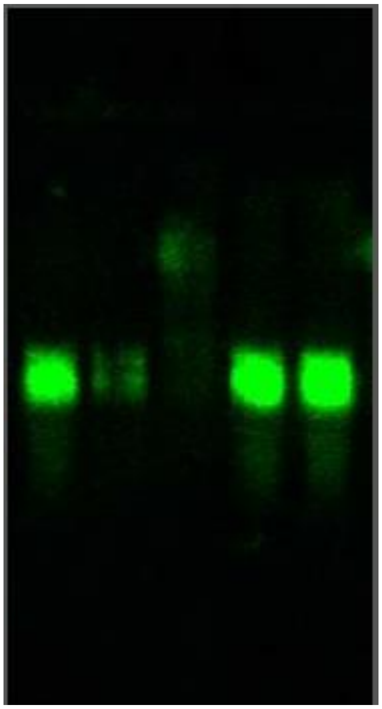

Dd2 WT-FP2

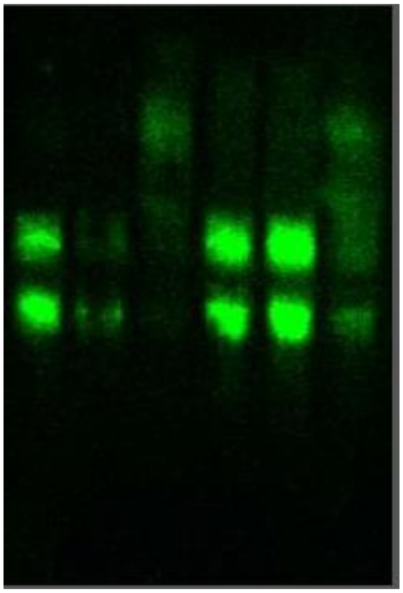

Dd2 WT-FP2-PNP

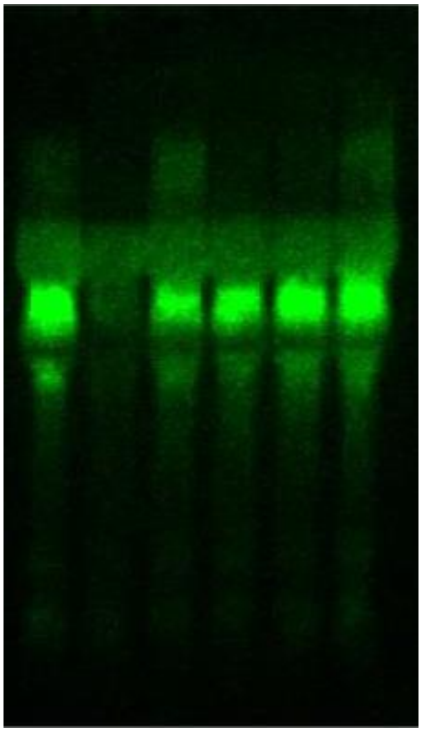

Dd2 Ra-FP2-CysPxn

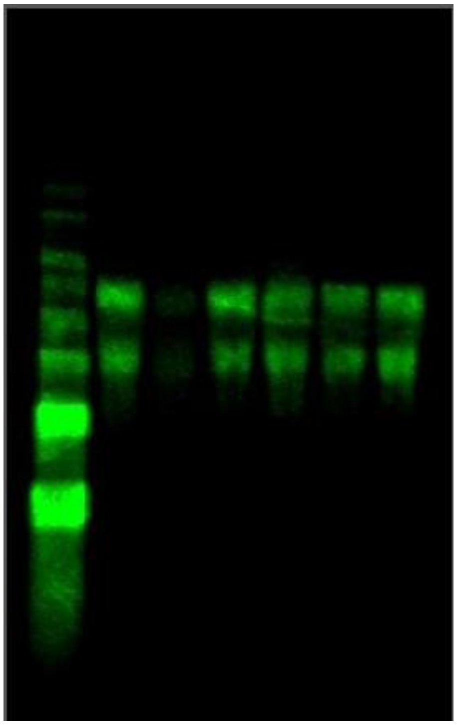

Dd2 Ra-FP2-PNP

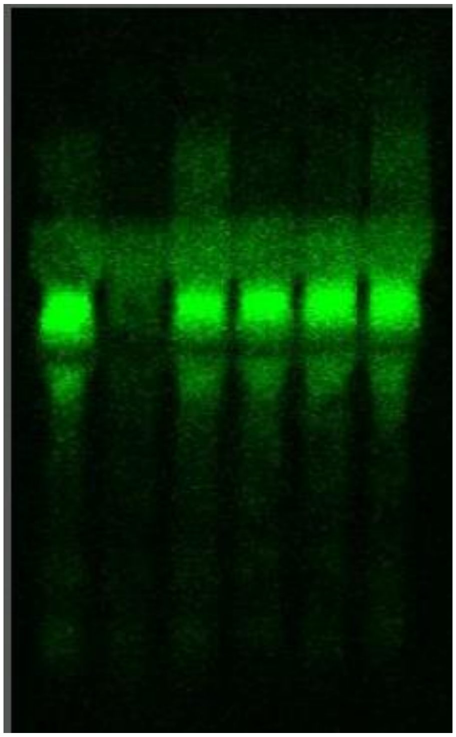

Dd2 Rb-FP2-CysPxn

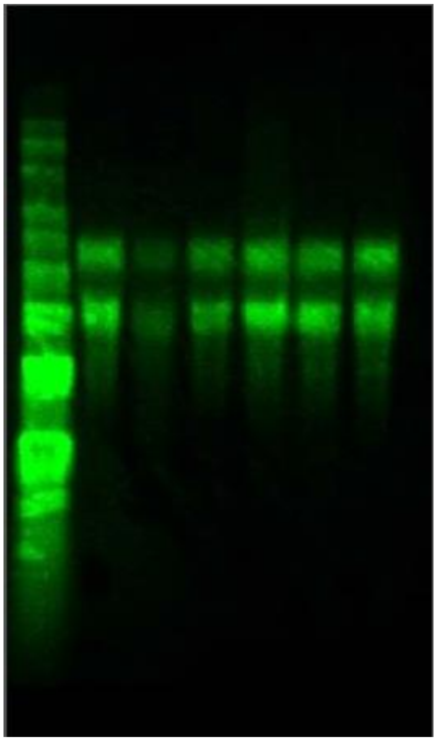

Dd2 Rb-FP2-PNP

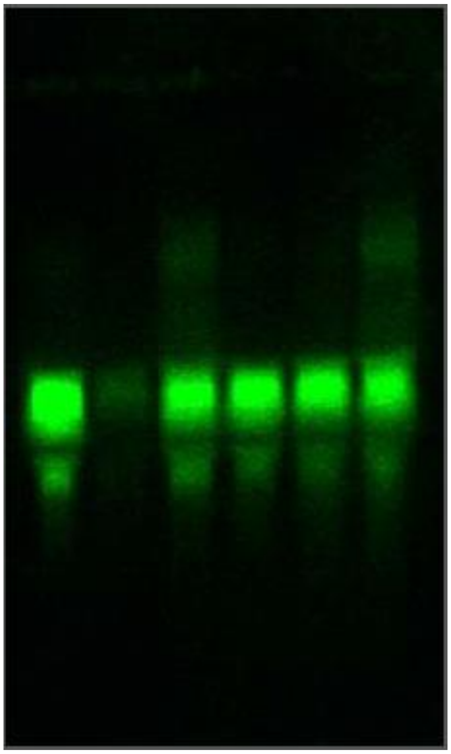

Dd2 D3cl.4-FP2-CysPxn

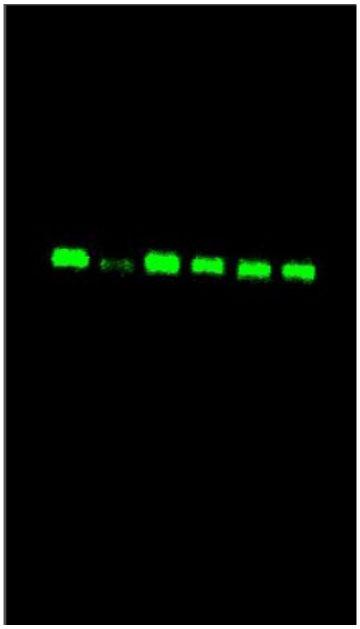

Dd2 D3cl.4-PNP

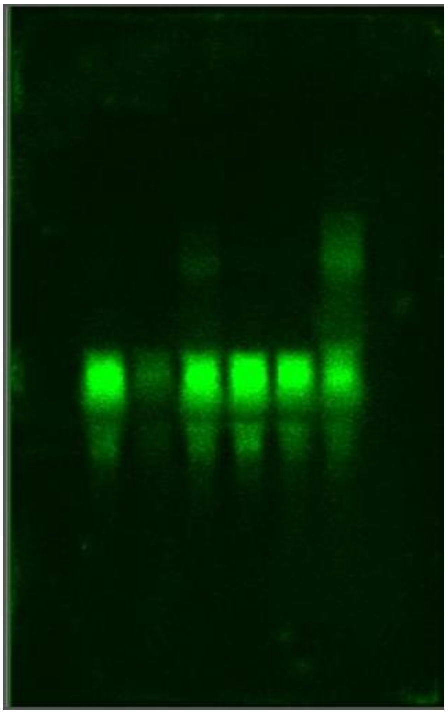

E1-FP2-CysPxn

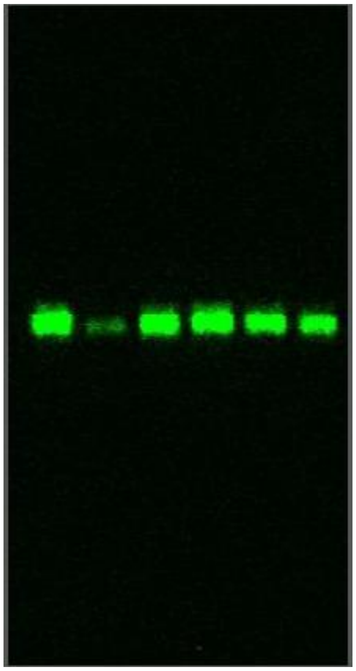

E1-FP2-PNP
